# New Materials and Techniques for Orthodontics

**DOI:** 10.3390/ma16051924

**Published:** 2023-02-25

**Authors:** Maria Francesca Sfondrini, Andrea Scribante

**Affiliations:** Unit of Orthodontics and Paediatric Dentistry, Section of Dentistry, Department of Clinical, Surgical, Diagnostic and Paediatric Sciences, University of Pavia, 27100 Pavia, Italy

Orthodontics is a specialty of dentistry dealing with the prevention, diagnosis, and treatment of mispositioned jaws and teeth. Orthodontic fixed therapy aims to reposition the patient’s teeth with specific appliances, usually brackets and wires. In order to ensure that correct treatment is provided, the bonding between the bracket and the enamel should be strong enough to support masticatory and shear forces. In fact, bracket failure is a frequent clinical concern in orthodontics both for the clinician and the patient. Accordingly, many efforts have been made by researchers in the last few years to improve bonding strategies as well as to test new innovative materials for clinical use with improved chemical and mechanical properties.

For instance, the current Special Issue entitled “New Materials and Techniques for Orthodontics” has been developed following on from the Special Issue entitled “Orthodontic Materials and Adhesive Interfaces”. In the first Special Issue, the use of a universal adhesive in total-etch mode was tested both in vitro and in vivo for the bonding of orthodontic fixed retainers, resulting in a good alternative to traditional orthodontic primers considering both shear bond strength (SBS) and the adhesive remnant index (ARI) [1]. Moreover, a fluoride release/rechargeable LiAl-F layered double hydroxide (LDH-F) orthodontic resin was tested for SBS and thermal cycling and resulted in better outcomes when compared to a control adhesive [2]. Further mechanical properties which have been tested for adhesives are represented by creep, hardness, and elastic moduli [3]. Finally, research on experimental orthodontic adhesives has been conducted to develop new products with antibacterial and remineralizing properties [4].

In addition to the study of orthodontic adhesives, research has also focused on bonding techniques, such as the indirect bonding technique [5], and the enamel surface roughness after lingual bracket debonding [6]. Moreover, other orthodontic materials have been the topic of extensive research. For instance, a glass ionomer cement modified using phytomedicine was proposed for its antibacterial properties, and it was tested as regards to its flexural strength, water sorption, and solubility [7]. Different composite materials have even been tested considering the correct reproduction of attachment shape and position [8].

The study of interface design and surface treatments also represents a relevant topic in the biomedical field, including orthodontics. A review was conducted to underline the antibacterial applications and mechanisms of metallic agents in both dentistry and orthopedics, indicating that much work still needs to be carried out to deepen our understanding of the antibacterial mechanisms and potential side-effects of metallic agents [9]. Biological interactions between the mouth and exogenous materials have also been studied considering the behavior of human oral epithelial cells grown in contact with aligners, with the latter showing no cytotoxicity [10].

The physical properties of orthodontic materials have been also evaluated in different studies. In particular, the mean shearing stroke frequency of different orthodontic bracket types and bonding agents under cycling loading was evaluated with the study in question showing that brackets of different types can be applied with different bonding techniques and that, in order to minimize the risk of hard tissue damage, ceramic brackets should be carefully bonded using the self-etching primary adhesive technique [11]. The compositional, microstructural, and mechanical characteristics of Ni-free orthodontic wires were compared to those of conventional stainless steel ones and they demonstrated similar properties thus making the former a promising alternative for patients with Ni supersensitivity [12]. Arch friction with conventional and self-ligating brackets has also been investigated, with higher static friction values being shown for conventional brackets [13].

One of the latest research topics in orthodontics with relevant clinical implications is represented by the effect of magnetic resonance imaging (MRI) and its effects on metallic brackets and wires. One study showed that, under MRI, orthodontic appliances present a low-temperature rise and no debonding risk, thus they are not recommended for the routine preventive removal of orthodontic appliances; the results instead suggested that this procedure be used only in the case of a void risk or potential interference in image quality [14].

In the current Special Issue, the included research topics are related to bacteriostatic coating for mini-implants [15], a 3D-printed orthodontic distalizer for unilateral class II treatment [16], and a retraction spring made of a new low elastic modulus material [17].

On the basis of these considerations, the authors would like to thank all of the clinicians and researchers who contributed relevant manuscripts to this Special Issue. In addition, the authors would also like to draw attention to future perspectives for further research. In particular, testing new, innovative materials for orthodontic use with remineralizing properties would certainly be of high impact; therefore, morphological and chemical studies would be welcomed [18]. The introduction of additional CAD/CAM 3D-printing materials and the evaluation of their clinical applicability in orthodontics would also be an interesting topic in the era of digital dentistry [19]. In addition, further studies on the metallurgical [20], bonding [21], flexural [22], mechanical [23], and biocompatibility [24] characteristics of different orthodontic biomaterials would also be welcomed in the future. Finally, the importance of the recent introduction of artificial intelligence [25] would be an interesting future research perspective as well.
